# The Strathclyde Evaluation of Children's Active Travel (SE-CAT): study rationale and methods

**DOI:** 10.1186/1471-2458-11-958

**Published:** 2011-12-30

**Authors:** David McMinn, David A Rowe, Shemane Murtagh, Norah M Nelson

**Affiliations:** 1 School of Medicine and Dentistry, University of Aberdeen, Aberdeen, UK; 2 School of Psychological Sciences and Health, University of Strathclyde, Glasgow, UK; 3 School of Culture and Lifestyle, University of Derby, Buxton, UK

## Abstract

**Background:**

The school commute is a prime opportunity to increase children's physical activity levels. However, active commuting has decreased over the past 40 years. Strategies that increase walking to school are therefore needed. Travelling Green (TG) is a school-based active travel resource aimed at increasing children's walking to school. The resource consists of a curriculum-based program of lessons and goal setting activities. A previous study found that children who received the TG intervention increased self-reported distance travelled to school by active modes and reduced the distance travelled by inactive modes. This study was limited by self-reported outcome measures, a small sample, and no follow-up measures. A more robust evaluation of TG is required to address these limitations. This paper describes the rationale and methods for such an evaluation of Travelling Green, and describes the piloting of various active commuting measures in primary school children.

**Methods/Design:**

Measures of active commuting were piloted in a sample of 26 children (aged 8-9 years) over one school week. These measures were subsequently used in an 18-month quasi-experimental design to evaluate the effect of TG on commuting behaviour. Participants were 166 children (60% male) aged 8-9 years from 5 primary schools. Two schools (*n *= 79 children) received TG in September/October 2009. Three schools (*n *= 87 children) acted as a comparison group, and subsequently received TG at a later date. Physical activity was measured using Actigraph GT1M accelerometers. Personal and environmental determinants of active commuting were measured via parent and child questionnaires, as were factors related to the Theory of Planned Behaviour and the construct of habit. Measures were taken pre- and post-intervention and at 5 and 12 months follow-up.

**Discussion:**

The piloted protocol was practical and feasible and piloted measures were reliable and valid. All study data, including 5 and 12 month follow-up, have been collected and processed. Data analysis is ongoing. Results will indicate whether TG successfully increases active commuting in a sample of Scottish school children and will inform future efforts in school active travel promotion.

## Background

Knowledge of the immediate and future health benefits of regular physical activity in children is well established [1], and it is known that even relatively small amounts of physical activity can have dramatic health benefits for children in high risk categories (e.g. obese, hypertensive) [2]. Physical activity promotion in child populations is therefore an important endeavour.

The journey to and from school has been identified as a prime opportunity to increase physical activity levels [3], and it has been shown that children who travel actively to school engage in more physical activity during the school commute than their inactive counterparts [4,5]. It has also been shown that children who actively commute are more active at other times of the day [6,7]. Furthermore, children who walk [8] and cycle [9,10] to school have higher levels of cardiovascular fitness than inactive travellers.

Despite the benefits associated with active school travel, active commuting to school across the western world has steadily decreased over the past 40 years [11-13]. Reasons for these declining trends are unclear, however contributing factors may include increased pressure on parents' time in the morning [14], perceived dangers on the route to school [15], and the convenience of using motorised transportation [16].

Several interventions have been designed to promote active school travel. Two examples of these are the Safe Routes to School (SRTS) programme and the Walking School Bus (WSB). The Safe Routes to School programme is a legislation-backed initiative in the United States whereby funding is provided to each state in order to address some of the barriers to walking and cycling to school. The majority of these funds are allocated to infrastructure changes such as traffic calming measures, street lighting, and cycle paths [17]. Funds are also used for non-infrastructure activities such as education and special events like walk and cycle to school days [18]. Studies that have been conducted to evaluate SRTS programmes have shown that children at schools taking part in the intervention increased walking, biking, and car-pooling [19], and that children who passed SRTS projects on their way to school were more likely to increase their walking or cycling [20].

A Walking School Bus is a group of supervised children that walks to school and picks up other children while travelling a predefined route. The WSB has become popular in many countries, particularly in New Zealand and Australia where it originated, and has been shown to successfully bring about increases in walking to school [21,22]. Moreover, the WSB is valued by those who coordinate and participate in them [23]. Although they have been shown to increase walking to school, several problems have been highlighted with the WSB, e.g. the need for parents or other volunteers to act as 'drivers', fading enthusiasm for the programme, and diminishing support from schools and councils [24].

Another intervention designed to promote walking to school is Travelling Green. This resource was designed in Scotland and takes the form of a 6-week project during which the class teacher delivers a series of curriculum-based lessons that cover various commuting and health-related topics. Children also set goals to walk to school on more days of the week by the end of the project. A small-scale evaluation of this intervention by McKee et al. found that children who took part in the project increased the distance travelled actively to school following the intervention, and decreased the distance travelled inactively compared with a control school [25]. Although the study showed positive results, there were several limitations. No objective measures of commuting behaviour or physical activity were used (results were based on self-reported distance travelled by mode). The sample was relatively small (one intervention school class, *n *= 31, and one control school class, *n *= 29), and there were no follow-up measures taken to determine if the intervention had any long term effects.

These limitations are not unique to the study conducted by McKee et al. Previous studies that have evaluated school travel interventions have had similar limitations, such as self-reported outcome measure [22,26], absence of control groups [19], and no follow-up measures [27]. There is therefore a need to conduct a robust evaluation of resources such as Travelling Green that addresses the limitations of previous evaluations of active school travel interventions. The study described in this paper aimed to achieve this by: (a) using objective commuting outcome measures; (b) using a larger sample; and (c) taking follow-up measures to assess any long-term effect of the intervention. This paper outlines the study rationale, aims, methods, and pilot work for the evaluation of Travelling Green.

## Method/Design

### Aim

This study was designed to pilot test several measures of active commuting in a sample of Scottish school children (*n *= 26) and to investigate the effect of Travelling Green on commuting behaviour in a sample of primary school children in Scotland (*n *= 166).

### Ethical approval

Ethical approval for all pilot and main evaluation procedures was granted by the University of Strathclyde Ethics Committee and all data collection was carried out in accordance with the Helsinki Declaration.

### Recruitment process

Recruitment for the pilot study was carried out in December 2008. Main study recruitment was carried out between February and June 2009. Permission to contact potential study schools was granted by all relevant local education authorities.

Study schools were sought from either end of the socioeconomic continuum, as defined by the Scottish Index of Multiple Deprivation (SIMD; http://www.scotland.gov.uk/Topics/Statistics/SIMD). The SIMD provides a relative measure of area level deprivation across 6,505 geographic data zones in Scotland. Area level deprivation is calculated using 38 indicators from the following 7 domains: Income, employment, health, education, housing, geographic access, and crime. This sampling method allows for an investigation of how school-level deprivation may influence commuting behaviour. Individual-level (home) SIMD was available by obtaining participants' home postcodes.

A purposive sampling approach was used to identify schools from the upper (most deprived areas) and lower (least deprived areas) quartiles of the SIMD. All potential study schools were located in urban areas, according to the Scottish Neighbourhood Statistics Urban Rural classification http://www.sns.gov.uk. Relevant council workers such as school travel coordinators, active schools coordinators, and road safety officers were contacted and asked to suggest potential study schools. Based on recommendations from these sources 11 schools were contacted to establish whether they would be interested in taking part in the study.

### Study population

Five schools agreed to participate in the study. Two schools were from areas in the low deprivation SIMD quartile; one intervention school (Int-LoDep) and one comparison school (Comp-LoDep). Three schools were from areas in the high deprivation quartile; one intervention school (Int-HiDep) and two comparison schools (Comp-HiDep: due to small numbers data from these two schools were combined to form one comparison group). It was not possible to randomly assign participants to the intervention or comparison group because schools had already scheduled the delivery of Travelling Green into their curriculum before agreeing to participate in the study, and so the intervention and comparison groups were somewhat pre-defined. However, all schools were similar in terms of already planning to implement Travelling Green at some point during the school year.

Participants were from primary 5 (typically ages 8-9 years) because this is the age group for which Travelling Green was designed. Descriptive characteristics of the study schools are displayed in Table 1. Study information sheets and consent forms were distributed to 232 children and their parents. Signed parent and child informed consent were obtained for 167 participants (72% response rate). Prior to the start of data collection one participant withdrew from the study, leaving a final sample of 166 participants. Children who did not provide consent took part in the intervention as part of the normal school curriculum, but no outcome measures were taken from these children.

**Table 1:** Study school characteristics

School	*n*	Deprivation level	% Employment deprived*	% of homes owned*	% Free school meals**
Int-LoDep	48	Low	2	97	2
Comp-LoDep	47	Low	3	98	5
Int-HiDep	31	High	19	39	37
Comp-HiDep	19	High	23	43	30
Comp-HiDep	21	High	14	52	26

*Scottish Neighbourhood Statistics, 2010 http://www.sns.gov.uk

**Free School Meals survey, 2009

http://www.scotland.gov.uk/Resource/Doc/920/0083583.xls

% Employment deprivation is the percentage of the working age population (16-64 for men and 16-59 for women) who are on the unemployment claimant count, are in receipt of Incapacity Benefit or Severe Disablement Allowance

### Study design

**Figure 1:**
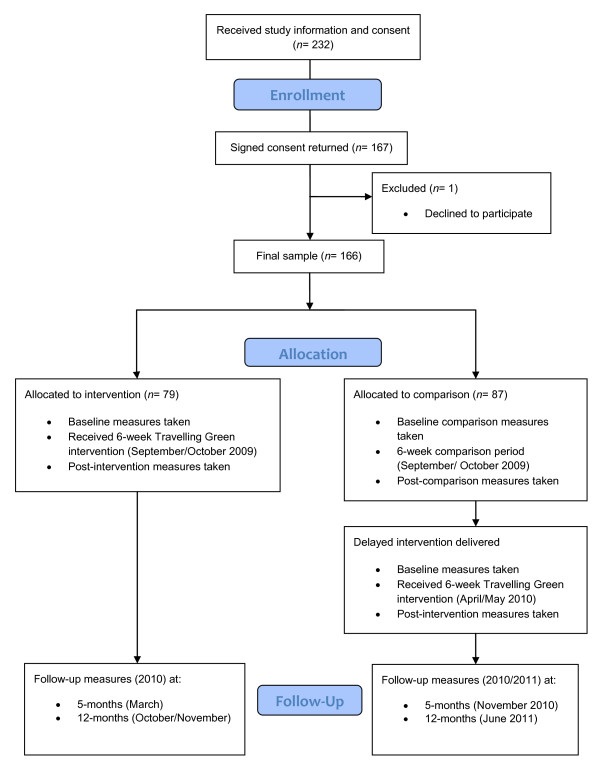
**Flowchart of study design and timeline**.

A quasi-experimental design was used. Two schools (Int-LoDep and Int-HiDep) received the Travelling Green intervention between August and October 2009. Measures of commuting behaviour (questionnaires, travel diary, and objective measures) were taken during 5 consecutive school days prior to starting the intervention and during 5 consecutive school days post-intervention. Three schools acted as comparisons during this period (Comp-LoDep and the two Comp-HiDep schools). The same measures were taken at these schools. The three original comparison schools received the intervention between April and June 2010, allowing for investigation of the effects of seasonality on the intervention. Furthermore, providing the intervention to the comparison schools meant that they would not miss out on any potential benefits of Travelling Green. Follow-up measures were taken at 5 and 12 months post-intervention at all schools to assess any lasting effect of the intervention on travel behaviour. Figure 1 shows the study design and timeline.

### The intervention

Travelling Green is a 6-week active commuting intervention that aims to increase children's walking to and from school. The project was designed for children aged 8-9 years, on the premise that children of this age are independent enough to travel to school alone, yet young enough to be enthused by a school-based project. The project is comprised of two components: A Teacher's Handbook and individual Pupil Packs for each child.

#### Teacher's handbook

The Teacher's Handbook contains introductory activities. These require children to consider their current school travel behaviours and think about the characteristics of a healthy journey to school. The main component of the Teacher's Handbook is a series of 13 lesson plans that cover a number of health-related and commuting topics. Topics include road safety, the heart and lungs, a healthy lifestyle, the Green Cross Code, and understanding the local environment. The lessons have been designed to link with the 'Curriculum for Excellence' (the Scottish national curriculum), and encompass key subject areas such as Health and Wellbeing, Social Studies, Expressive Arts, Technologies, and Languages. Lessons can be used flexibly, as and when the teacher feels appropriate, and can therefore be chosen to link with other topics being covered in the curriculum.

#### Pupil pack

The Pupil Pack contains the following elements: (a) a pupil information guide describing the project; (b) a *My Travel Challenge *booklet in which children set goals to travel actively on more days as the project progresses; (c) two wall charts on which children record how they travel to school each day, and how they travel home each day; and (d) two high visibility reflective stickers that can be attached to school bags or clothing.

Travelling Green was designed by West Dunbartonshire council in collaboration with NHS Greater Glasgow, and has been funded by the Scottish Government to be provided to every school in Scotland. Distribution of the resource is being coordinated by the sustainable transport organisation Sustrans. A member of the research team (DM) trained the teachers at each school so that they were able to deliver the project.

### Measures

#### Actigraph GT1M

The Actigraph GT1M (ActiGraph, Pensacola, FL) physical activity monitor is 3.8 × 3.7 × 1.8 cm and contains a uni-axial accelerometer. Devices in this study were attached to an elastic belt and worn on each participant's right hip. Vertical bodily accelerations are converted participant's into activity counts, which are monotonically related to magnitude of acceleration i.e. as activity intensity increases, so do activity count values. Counts are recorded over a pre-selected period of time (epoch), ranging from 1 s to 1 min. At the end of each epoch activity counts and steps are summed and stored. Validated cutpoints can be applied to the activity count data to determine time spent in different activity intensities. The GT1M is a widely used measure of physical activity in the research community, and has been validated for use in children against indirect calorimetry in both laboratory [28] and free-living [29] conditions.

The GT1M devices were initialised, and data were downloaded using Actilife data analysis software (version 3.2.2; ActiGraph, Pensacola, Florida, USA). Initialisation involves setting the time, date, epoch length, and assigning a file name. In this study, 5-s epochs were used (this is the shortest epoch length that allows data storage over 5 days). Prior to initialising the devices, the computer used for initialisation was time synchronised with a digital watch to allow for accurate recording of participants' morning arrival times at school. This was important for subsequent data processing.

#### New lifestyles NL-1000

The NL-1000 (New Lifestyles, Inc., Lee's Summit, MO) is a new type of pedometer that uses a piezoelectric mechanism similar to an accelerometer. The NL-1000 costs considerably less than an accelerometer and does not require initialisation or downloading. The device measures 6.4 × 3.8 × 2 cm, and in this study was attached to the elastic belt alongside the GT1M. The NL-1000 records steps and time spent in different activity intensities, and these data are read from a digital display. The device also features an automatic 7-day memory function, whereby daily activity (steps and MVPA time) are stored during the night and the display is reset for the following day.

The NL-1000 can be set to record time spent in different activity intensities. There are 9 discrete thresholds available, each corresponding to one of three activity intensities (1-3 light, 4-6 moderate, and 7-9 vigorous). Each threshold corresponds to a metabolic equivalent (MET) value, ranging from 1.8 to 8.3 METs. The lower and upper bounds of the intensity level can be changed to suit research needs. For example, if a researcher is concerned only with vigorous activity then the threshold can be set at a higher level. The default setting is 4-9, i.e. moderate-to-vigorous physical activity (MVPA). Any activity performed at or between these bounds will be added to the activity time. The MVPA threshold for the pilot study was set at level 3 (2.9 METs) and then increased to level 4 (3.6 METs) for the main evaluation study. The threshold was increased following suggestions that a value greater than 3 METs should be used as the MVPA threshold in children, to account for their higher resting energy expenditure [30].

#### Child questionnaire

A child school travel questionnaire was designed specifically for this study. The questionnaire gathered information about children's: (a) demographics; (b) usual mode of travel to and from school; (c) stage of behavioural change related to walking to school; (d) perceived barriers, facilitators, and benefits to walking to school; (e) preferred mode of travel to school; (f) self-efficacy for various commuting-related tasks; (g) perceptions of the local area; and (h) commuting behaviour in relation to the Theory of Planned Behaviour (TPB) and the construct of habit.

Most items in the questionnaire were taken from a pupil questionnaire located in the introductory activities of the Teacher's Handbook in the Travelling Green resource. A question related to perceptions of the local area was adapted from an item in the Traffic and Health in Glasgow Questionnaire [31]. TPB items concerned with attitude, subjective norm, perceived behavioral control, and intention were adapted from previously used items [32]. The Self-Report Habit Index [33] was used to measure habit in relation to walking, and car and bus use as school commuting modes. One question investigating participants' self-efficacy for certain active commuting tasks was developed specifically for this study. Questionnaire item response formats were a mix of tick box and Likert scale. Validity and reliability evidence for the child questionnaire was established during the pilot study, results of which are presented later.

#### Parent questionnaire

The parent questionnaire elicited similar information to the child questionnaire, but answered from the parent's perspective. In addition, the parent questionnaire gathered data on: (a) the child's health status and ethnicity; (b) parent's age and various socioeconomic indicators such as car ownership and employment status; and (c) street connectivity in their area. Validity and reliability evidence for the parent questionnaire was not investigated, however the questionnaire was compiled using items from the following existing questionnaires: (a) Pupil questionnaire in the Travelling Green resource; (b) the Traffic and Health in Glasgow Questionnaire [31]; and (c) the 'Active Where' Parent-child Survey 1 [34,35]. Items investigating factors related to the TPB and habit were not included in the parent questionnaire.

#### Travel diary

Travel diaries were used to gather information about the trip home from school. Diaries asked participants what time they arrived home, how they travelled home, and if they went via another location on the way home. Children were asked to complete the diaries retrospectively (i.e. the next morning when they arrived at school) to achieve a higher response rate. Home times reported on the diaries were later used to inform Actigraph GT1M data processing. Validity evidence for the travel diary is reported in the pilot study results.

### Pilot study

#### Pilot aims

The pilot study had two aims. Firstly, to assess the practicality and feasibility of using the previously described procedures and measures in a school setting. Secondly, to establish validity and reliability evidence for (a) the NL-1000, (b) the child questionnaire, and (c) the travel diary, for use in school travel research, with a view to using these measures to evaluate Travelling Green.

#### Pilot method

A cross-sectional design was used, with a sample of 26 primary 5 pupils (8-9 years) from a school in the west of Scotland. These participants were different to those who participated in the main Travelling Green evaluation.

Pilot data collection was conducted over one school week (Monday to Friday). On the Monday participants completed the child questionnaire under the guidance and supervision of the research team. Participants were then shown how to wear the belt (with attached activity monitors) correctly and were asked to put their belt on at 3 pm before travelling home. Travel diaries were also administered.

Participants were asked to wear the belt when travelling to and from school for the duration of the week. On arrival at school each morning a member of the research team recorded each participant's arrival time and NL-1000 steps and time spent in MVPA (minutes and seconds). Participants removed their belts and one of the researchers reset the NL-1000s and stored the belts with attached monitors in the classroom throughout the day. This was done so that when the belts were worn on the trip home from school data collected would be relevant to the commute only, and this data would later be saved to the NL-1000 device memory (allowing home commute data to be recorded the following morning from the device memory). Immediately before travelling home from school participants put their belt back on. Participants had been asked to remove their belts as soon as they arrived home. This resulted in the GT1M output displaying a consecutive sequence of zeros when downloaded, thus providing an accurate record of home arrival time - to subsequently be compared to travel diary-reported home time. The following morning one of the researchers recorded the home trip data from the NL-1000 memory. This protocol was repeated on each day of data collection. Activity monitors and travel diaries were collected on the Friday. A second (retest) child questionnaire was administered one week after the first administration.

Test-retest reliability for the child questionnaire was established by calculating percent agreement between items (nominal level data). and using Spearman's rho (ordinal level data). Reliability of the self-efficacy item designed specifically for this study was investigated using an intraclass correlation coefficient (ICC) from a 1-way ANOVA model, adjusted for a single measure.

Validity evidence for the travel diary was established by comparing known home arrival times (determined using the GT1M data) to diary reported home arrival times using Wilcoxon signed ranks test and Spearman's rho (ρ).

Validity of the NL-1000 was investigated by comparing step and MVPA data to data recorded by the GT1M (criterion measure) during the school commute. GT1M steps and time spent in MVPA were calculated for the journeys to and from school using Microsoft Excel 2007 (Microsoft Corp, Redmond, WA). Activity at or above a threshold of 3 METs was considered to be of moderate intensity. A threshold of 3 METs was used as this corresponded to the MVPA threshold used in the NL-1000. Age specific cut-points based on a previously developed [36] and published [37] MET prediction equation were used to establish time spent in MVPA. Morning and afternoon commute data were combined to create total commuting steps and total commuting MVPA for each participant for both instruments (NL-1000 and GT1M). Differences between GT1M and NL-1000 data (steps and MVPA) were compared using Wilcoxon signed-rank test, and correlation between instruments was tested using Spearman's rho. Non-parametric tests were used because NL-1000 data were non-normally distributed (see Table 2).

**Table 2:** NL-1000 and GT1M descriptive statistics

	Mean	SD	**Min**.	**Max**.	**Skew**.	**Kurt**.
NL-1000 MVPA (Sec)	485	362	80	1720	2.10	6.10
NL-1000 Steps	1302	805	376	3999	2.00	5.60
GT1M MVPA (Sec)	584	328	152	1485	1.20	1.43
GT1M Steps	1002	619	275	2719	1.30	1.71

#### Pilot results

The majority of questionnaire items had test-retest agreement of above 70%, which was deemed acceptable. The two items on which correlational analysis were carried out demonstrated high correlations, *ρ *= .87 and *ρ *= .76, and the self-efficacy item had high reliability, *ICC *= .93, single measure = .86. The Theory of Planned Behaviour item and the Self-Report Habit Index item have been used previously with children of a similar age to the sample in this study [32,38,39].

No significant differences were found between the home arrival times reported on the travel diaries to the known arrival times (mean error = 6 min, *z *= .56, *p *≥ .05). Furthermore significant moderate correlations were found between measures (*ρ *= .42, *p *< .01).

Descriptive statistics for NL-1000 and GT1M data are displayed in Table 2. The NL-1000 significantly underestimated time spent in MVPA during the school commute compared to the GT1M (mean difference = 99 s, *z *= -3.08, *p *< .01), and significantly overestimated steps compared with the GT1M (mean difference = 300 steps, *z *= -4.02, *p *< .01). However, the NL-1000 and GT1M were highly correlated for measuring MVPA (*ρ *= .95, *p *< .01) and steps (*ρ *= .96, *p *< .01) during the school commute. Furthermore, according to Cohen's *d *[40] effect size, the differences between instruments in MVPA and step estimates were small to moderate (*d *= 0.29 and *d *= 0.42 respectively).

#### Pilot conclusions

Participants generally understood the questions in the child questionnaire, and when a child did not understand a word or phrase a member of the study team was available to help. No feasibility issues were reported for travel diary or activity monitor protocols, however it was common for participants to forget to put their belt on before leaving for school (data were lost on 18% of days this way).

Results from the pilot study indicate that the child questionnaire and travel diary are valid and reliable tools for use in travel-related research with children aged 8-9 years. Adequate validity evidence was provided for the NL-1000 as a measure of commuting-related physical activity. Although the differences between the NL-1000 and GT1M estimates of MVPA and steps were small to moderate, it was decided to continue to use the NL-1000 alongside the GT1M in the main evaluation study in order to generate additional validity evidence for the NL-1000.

### Main evaluation study

#### Procedures

These procedures refer to data collected pre- and post-intervention, and at 5- and 12-months follow-up. On the Monday of each data collection week members of the research team went to the relevant school to administer the commuting measures. The research team distributed the questionnaires (child and parent) and travel diaries. Participants were asked to store their travel diary in a safe place in the classroom and complete each morning. Parent questionnaires were to be taken home and returned during the week; the same parent who completed the questionnaire at baseline was asked to complete the questionnaire post-intervention, and at 5- and 12-month follow-up. Participants sat in small groups to complete their questionnaire, and each group was supervised by a member of the research team. Several research assistants had been trained to assist with data collection, and so there was typically a ratio of 4 children to each research assistant. Participants were given their belt (with attached activity monitors) on completion of their questionnaire. The time that the activity monitors were distributed was recorded for GT1M data processing purposes.

Participants were asked to wear their activity monitors during waking hours, and only to remove them during sleep, swimming, bathing, and contact sports. Participants were also asked to approach one of the research team in the school playground each morning on arrival at school to have their arrival time and NL-1000 steps and MVPA recorded. The protocol for the main evaluation study differed slightly from the pilot study in that participants were asked to wear the activity monitor across the whole day in the main evaluation study, rather than only wearing it during commuting time, as in the pilot study. Participants only wore the monitors during commute time in the pilot study to (a) allow a known home arrival time to be established for comparison with the travel diary reported home time, and (b) to allow the validity of the NL-1000 to be investigated specifically during active commuting.

On the Friday of data collection participants' activity monitors, parent questionnaires, and travel diaries were collected. This was done after the time of day that the activity monitors had been handed out on the Monday to allow Monday afternoon data to be combined with Friday morning data in order to create a composite day. GT1M data were downloaded on Friday evening and NL-1000 daily MVPA and step totals were retrieved from the device memory and entered into a master data sheet. Questionnaire and travel diary data also were entered into the master data sheet. GT1Ms were recharged and initialised over the weekend ready for the next week of data collection (there was not enough equipment or researchers to test schools simultaneously).

#### Data management

Electronic data were stored on a password protected computer, and hard copy data (i.e. questionnaires and travel diaries) were kept in a locked filing cabinet. Participants were assigned identification numbers to protect their identity.

#### Actigraph GT1M data processing

Initially, non-wear GT1M data were deleted. These were: (a) data before the activity monitors were distributed on the Monday, and after collection on the Friday; (b) data between the hours of 23:30 and 05:30 (i.e. sleeping time); and (c) data on days when the participant was absent or had forgotten to wear their belt (according to written records). Monday afternoon data were then merged with Friday morning data to create a composite day, resulting in 4 full days of data. Steps and time spent in MVPA were then calculated for three time periods: (a) morning commute, (b) afternoon commute, and (c) full day. Morning commute was defined as being from 05:30 to the time the child arrived at school (as recorded by the study team). Afternoon commute data were processed differently depending on the mode of travel reported on the travel diary. If the participant reported walking home, data were analysed from 15:00 (end of school day) to the self-reported home time. If the reported home time was before 15:15 then data were analysed up to 15:15. Data for participants who travelled home inactively were analysed from 15:00 to 15:15. Therefore each participant was credited with a home commute time of at least 15 min. The individualised approach used to calculate afternoon commute time for walkers and non-walkers was taken to avoid unfairly biasing walkers, who often take longer to commute than children who travel by car. If travel diary data were unavailable, then afternoon commute activity was deemed as missing and was later replaced. Full day was defined as being between 05:30 and 23:30. Steps were calculated using the 'sum' function in function in Excel. MVPA was defined as any activity at or above 4 METs, derived using a previously published MET prediction equation [37], adjusted for 5 s epochs. Cutpoints ranged from 136-171 counts/5 s. Two large studies have previously used this equation to establish cutpoints equivalent to different activity intensities [41,42], and so using these cut-points allows for comparative data to be generated. The MVPA threshold was set at 4 METs in the main study because of recent suggestions that a threshold greater than 3 METs is more appropriate for children [30], to adjust for their higher resting energy expenditure [43]. Following data processing, step and MVPA data were pasted into a master Excel file ready for missing data replacement. No wear time criterion was used in this study. It was assumed that if participants arrived at school wearing their GT1M there would be at least 8 h of data collected (6 school-day hours and approximately 1 h before and 1 h after school). This is similar to the 10 h per day wear time criterion used in previous studies with children [41,42]. Furthermore, data for days on which participants forgot to wear their belt, or were absent from school, were deleted based on written records.

#### Data checking and replacement

Initially, data were checked for inputting errors. A random selection of participants' hard copy data (i.e. questionnaires and travel diaries) were read aloud by one of the research team while another member of the team visually inspected the data sheet for agreement. 10% of data were checked. Data inputting errors were < 5%. Single data entry was used in this study as it has been shown that double data entry considerably increases data inputting time [44] and may not materially improve the quality of the final data set [45,46]. Furthermore, range checks on each variable were carried out during and after data entry to identify and correct errors that may have affected the final results and conclusions.

Missing data analyses were then carried out to establish type and percentage of missing data. Written records from a data collection diary were consulted to identify days on which participants had forgotten to wear their belts or had been absent from school. NL-1000 data for such days were deleted (GT1M data for these days had previously been deleted during data processing). Participants with missing questionnaire (both child and parent) and travel diary data were also identified.

Pre-intervention, GT1M and NL-1000 data were missing for 78 of 664 days (11.7%), 12 participants (7.2%) had missing GT1M and NL-1000 data for the whole week, 11 participants (6.6%) had completely missing questionnaire data, 28 parents (17.0%) did not return their questionnaires, and 10 participants (6.0%) lost their travel diary.

Post-intervention, GT1M and NL-1000 data were missing for 169 of 664 days (25.5%), 13 participants (7.8%) had missing GT1M and NL-1000 data for the whole week, 3 participants (1.8%) had completely missing questionnaire data, 58 parents (35.0%) did not return their questionnaires, and 24 participants (14.5%) lost their travel diary. 3 participants (1.8%) had no GT1M and NL-1000 data for both pre- and post-intervention. This information was used to inform data replacement.

Outlying data were identified for daily GT1M steps using previously published guidelines [47]. Daily steps were classed as outlying if values were < 1,000 or > 30,000 steps/day. No outlying data were found pre-intervention. Post-intervention, three participants had daily step values < 1,000. These data points were deemed to be unrepresentative of the population in question and were therefore deleted and later replaced, as were corresponding daily MVPA data.

Missing data were replaced before any statistical analyses were performed. Missing data were diverse in nature due to the multiple outcome variables being measured. Various data replacement techniques were therefore used. Team meetings were held to identify and discuss available data replacement techniques. These discussions led to the most appropriate replacement techniques being selected for the different types of missing data. A complete report of missing data procedures is available from the corresponding author.

Individual missing step and MVPA data points were replaced using an individual information centered (IIC) technique [48]. This involved replacing a missing data point with the mean value of remaining data points for a given individual. This technique has been shown to be more accurate than group information based approaches e.g. using a group mean to replace data for an individual [49].

If a single questionnaire item within a scale was missing, IIC was used. If a whole scale was missing, data were replaced using the participant's corresponding data from the other data collection week. For example, if a participant was missing a whole scale from the post-intervention questionnaire, these data were replaced using data from their pre-intervention questionnaire. This replacement technique was also used for participants missing a whole week of data (either all of their activity monitor data or questionnaire data). This technique assumed no change from pre- to post-intervention, and protected against committing a type 1 error. This was particularly important for data from participants who received the intervention. Some data were deemed inappropriate to replace and were therefore left missing, for example questions about participant's preferred mode of travel, or preferred travel companion.

Group mean replacement (based on school and gender) was used to replace data for three participants who were missing both pre- and post-intervention GT1M data. This data loss was due to a combination of lost devices and device malfunction.

Following data replacement, data were exported from the Excel spreadsheet into an SPSS 17.0 data file ready for analysis.

#### Data analysis and sample size calculations

Descriptive statistics will be used to summarise the sample characteristics. The effect of Travelling Green on children's walking to school will be investigated using a mixed two-way factorial ANOVA, using commuting steps as the primary outcome measure.

G-Power (version 3.1.2) was used to calculate the required sample size for the primary outcome of commuting steps. The statistical test was set at F-test ANOVA, effect size f (Cohen's *f*) was set at 0.15 (medium), Alpha level was set at 0.05, for a power of 80%, using a within-between groups design. There was a high correlation among repeated measures (*r *= 0.73). The total required sample size based on these parameters was 50 (25 in each group).

Data generated through this study will be used to answer several other research questions concerned with: (a) the influence of seasonality on Travelling Green effectiveness; (b) the long term effect of Travelling Green on commuting behaviour; (c) the personal and environmental determinants of active commuting in children; (d) parent's role in children's choice of travel mode; (e) the moderating effect of socioeconomic status and deprivation level on school travel behaviour change; and (f) the role of habit in children's school commuting behaviour.

## Discussion

This paper set out the study rationale and methods for the Travelling Green study, which had the primary aim of (a) investigating the immediate and long-term effects of Travelling Green on walking to school in a large sample of Scottish children, and (b) pilot testing various measures of active commuting in the school setting. Additional research questions (identified above) will be answered using data generated from this study.

The piloted active commuting measures were found to be reliable and feasible for use in the primary school population, and were therefore used in the main evaluation of Travelling Green. Results from the main study evaluating Travelling Green will help to inform the research community of strategies that may or may not successfully increase children's walking to school.

All data (i.e. pre, post, 5 and 12 month) have been collected and processed. Several data collection issues were encountered. Children regularly forgot to put on their belts before going to school, and equipment loss was common. This posed obvious constraints including data loss and the need to obtain additional equipment. It is interesting that children forgot to wear their belts more frequently during post-intervention data collection, and equipment loss was also greater during this time. This suggests that participants may have become less enthused by the study over time, and the novelty of wearing the belts may have worn off as the study progressed. Incentives for children to wear their belts and return them at the end of data collection (small frisbees and wrist bands) were introduced at 5 and 12 month data collection. This approach improved adherence to protocols and similar methods are therefore recommended in order to improve compliance in future physical activity research with children.

Conducting research in the school environment can present unforeseen challenges, and several difficulties were encountered during this study. These included difficulties contacting and communicating with relevant school staff due to their busy work schedules, for example. It was also challenging to create an atmosphere conducive to conducting controlled research in the school environment, for example one of the study schools had open-plan classrooms where children were easily distracted by activities going on in surrounding classes. These issues were not necessarily the fault of any one individual or group, but are somewhat inherent obstacles of school-based research. Similar issues have previously been reported by other school-based researchers [50,51]. Researchers proposing to conduct a school-based study should ensure that they are well prepared and have efficient protocols in place to fit their study into a busy school schedule. Additionally, researchers should plan for unexpected events and have contingency plans in place.

Methodological issues were encountered that relate specifically to conducting school commuting research, and issues that relate more generally to conducting physical activity research. Previous school travel studies have used a variety of definitions to categorise an individual as either an active or inactive commuter. These include self-reported usual mode of travel [8], parent proxy reported usual mode [52], mode of travel used on the day of survey [19], number of trips by mode over the past week [53], and direct observation [54]. These methodological differences make it difficult to compare findings between studies, and a consensus on one method of defining commuting mode would be helpful. In the present study, usual mode of travel to and from school was provided by the parent and the child. Parent responses may be more accurate as they responded without the presence of researchers (i.e. in their own home) and therefore may be unaffected by social desirability bias.

Another methodological challenge encountered in this study was related to accelerometer use, specifically in choosing one of the many available cut-points to define MVPA. Available 1 min cutpoints for 3 METs in children range from 615 to 3200 cpm [55,56], and no consensus has been reached in the published literature as to which cut-point is most appropriate [30,57]. Similar to the issue of defining an active commuter, reaching a consensus on appropriate accelerometer cut-points in children would allow for comparisons to be made between studies. In the present study, age specific MVPA cut-points were calculated for 4 METs using a MET prediction equation developed by the Freedson research group [36] and published by Trost et al. (2002) [37]. This equation was developed using data from 80 participants aged 6-18 year olds during treadmill walking and running and has previously been used in two population-based studies with children to determine time spent in MVPA [41,42]. A limitation of this equation is that it was not developed under free-living conditions.

The current study has several limitations and strengths. One limitation is the lack of randomisation, however as previously stated randomisation was not possible due to existing school schedules. It is acknowledged that randomisation would protect against any underlying systematic differences between groups. A lack of health-related outcome measures such as BMI, heart rate, or cardiovascular fitness may also be seen as a study limitation. However, it is unlikely that a change in any of these outcome variables would be observed after 6 weeks. Moreover, the primary goal of Travelling Green is to increase walking as a commuting mode, not to directly change health outcomes. Another limitation is the absence of comparison groups for the duration of the study i.e. no comparison for 12 month follow-up measures because the comparison group received the Travelling Green intervention at 5 months. It was felt that it would be unethical to postpone the delivery of Travelling Green longer than necessary; therefore a minimal control period was used. Finally, the study sample is only representative of children from either end of the socio-economic continuum (i.e. high and low deprivation). Study results should therefore only be generalised to children from these populations.

This study has several strengths. Previous studies that have investigated the effect of school commuting interventions have often lacked control groups, have used self-reported outcome measures, and have failed to obtain follow-up measures. The present study addresses each of these limitations by using a quasi-experimental design, by obtaining objectively measured physical activity data, and by taking follow-up measures at 5 and 12 months post-intervention. In addition, to the authors' knowledge, this is the first study to accurately establish activity levels during commute time using accelerometry. Previous studies using accelerometry have defined the school commute using segments of time before and after school, for example 08:00-09:00 and 15:00-16:00 [5,6], and thus have inevitably captured activity that was not related to the commute (e.g. physical activity in the playground before the start of school). The accurate measurement of commuting behaviour in this study reduces error and will provide a more accurate picture of the contribution of the school commute to daily physical activity.

In conclusion, the Travelling Green study will create important evidence for the possibility of increasing walking to and from school using a school-based active travel intervention. This information will contribute to the growing evidence base of strategies used to curb the declining trends in walking to school. Additionally, the pilot work for the Travelling Green study provides valuable reliability and validity evidence for several active commuting procedures and measures that can now be used with confidence in future commuting-related studies.

## Competing interests

The authors declare that they have no competing interests.

## Authors' contributions

DAR and NMN conceived the study design. All authors contributed to the development of the questionnaires and final study protocol. DAR was the principal investigator and led the study. DM and SM collected the data. DM drafted the manuscript and all authors provided feedback and approved the final version.

## Pre-publication history

The pre-publication history for this paper can be accessed here:

http://www.biomedcentral.com/1471-2458/11/958/prepub
